# Beta-band power classification of go/no-go arm-reaching responses in the human hippocampus

**DOI:** 10.1088/1741-2552/ad5b19

**Published:** 2024-07-15

**Authors:** Roberto Martin del Campo Vera, Shivani Sundaram, Richard Lee, Yelim Lee, Andrea Leonor, Ryan S Chung, Arthur Shao, Jonathon Cavaleri, Zachary D Gilbert, Selena Zhang, Alexandra Kammen, Xenos Mason, Christi Heck, Charles Y Liu, Spencer Kellis, Brian Lee

**Affiliations:** 1 Department of Neurological Surgery, Keck School of Medicine of USC, University of Southern California, Los Angeles, CA, United States of America; 2 USC Neurorestoration Center, Keck School of Medicine of USC, Los Angeles, CA, United States of America; 3 Department of Neurology, Keck School of Medicine of USC, University of Southern California, Los Angeles, CA, United States of America; 4 Keck School of Medicine of USC, University of Southern California, Los Angeles, CA, United States of America; 5 Viterbi School of Engineering, University of Southern California, Los Angeles, CA, United States of America

**Keywords:** beta-band power modulation, stereotactic electroencephalography (SEEG), human hippocampus, center-out go/no-go task, arm-reaching movements (ARMs), principal component analysis (PCA), discriminant analysis (DA)

## Abstract

*Objective.* Can we classify movement execution and inhibition from hippocampal oscillations during arm-reaching tasks? Traditionally associated with memory encoding, spatial navigation, and motor sequence consolidation, the hippocampus has come under scrutiny for its potential role in movement processing. Stereotactic electroencephalography (SEEG) has provided a unique opportunity to study the neurophysiology of the human hippocampus during motor tasks. In this study, we assess the accuracy of discriminant functions, in combination with principal component analysis (PCA), in classifying between ‘Go’ and ‘No-go’ trials in a Go/No-go arm-reaching task. *Approach.* Our approach centers on capturing the modulation of beta-band (13–30 Hz) power from multiple SEEG contacts in the hippocampus and minimizing the dimensional complexity of channels and frequency bins. This study utilizes SEEG data from the human hippocampus of 10 participants diagnosed with epilepsy. Spectral power was computed during a ‘center-out’ Go/No-go arm-reaching task, where participants reached or withheld their hand based on a colored cue. PCA was used to reduce data dimension and isolate the highest-variance components within the beta band. The Silhouette score was employed to measure the quality of clustering between ‘Go’ and ‘No-go’ trials. The accuracy of five different discriminant functions was evaluated using cross-validation. *Main results.* The Diagonal-Quadratic model performed best of the 5 classification models, exhibiting the lowest error rate in all participants (median: 9.91%, average: 14.67%). PCA showed that the first two principal components collectively accounted for 54.83% of the total variance explained on average across all participants, ranging from 36.92% to 81.25% among participants. *Significance.* This study shows that PCA paired with a Diagonal-Quadratic model can be an effective method for classifying between Go/No-go trials from beta-band power in the hippocampus during arm-reaching responses. This emphasizes the significance of hippocampal beta-power modulation in motor control, unveiling its potential implications for brain–computer interface applications.

**Abbreviations**
ARM: Arm-reaching movement
SEEG: Stereotactic electroencephalography
fMRI: Functional magnetic resonance imaging
PCA: Principal component analysis
LFP: Local field potential
aDBS: Adaptive deep brain stimulation
BCI: Brain–Computer Interface
DA: Discriminant Analysis
LDA: Linear discriminant analysis
QDA: Quadratic discriminant analysis
IRB: Institutional Review Board
USC: University of Southern California
ITI: Inter-trial interval
IIS: Interictal spike
IQR: Interquartile range
CV: Cross-validation
PC1: Principal component 1
PC2: Principal component 2
STN: Subthalamic nucleus

## 1. Introduction

Although the hippocampus is typically known for its role in episodic memory [1], spatial navigation [2], and motor sequence memory consolidation [3], advances in stereotactic electroencephalography (SEEG) and functional magnetic resonance imaging (fMRI) have allowed researchers to investigate a broader range of roles of its function, such as those involved in motor control [4, 5]. Recently, several studies have suggested the presence of coupling between the hippocampus and various cortical structures associated with movement during learning and adaptation [6], as well as during paced motor tasks [7]. These cortical structures include areas in the sensorimotor cortex, which have been shown to produce task-specific motor output following rapid integration of different sensory modalities [8]. The presence of strong effective connections between these structures even in the absence of motor learning implies that the hippocampus is heavily linked to the control of volitional movement [7].

The detection and examination of beta-band power through SEEG recordings has played a pivotal role in understanding the neural correlates of movement and behavior [9]. Previous research has extensively studied the temporal evolution of beta-band power, which consists of neural potentials oscillating at frequencies of 13–30 Hz [10–14]. Although the beta-band is typically defined by this frequency range, some studies have considered signals as low as 12 Hz [15, 16] and 12.5 Hz [17, 18], as well as signals as high as 35 Hz [19, 20] to be included in the beta-band. Despite this variation, the operative range of beta-band activity specifically as it pertains to motor function is generally agreed to be between 13–30 Hz [11, 12, 21]. In our previous research, we have used this or a smaller range of the beta-band in motor control studies of the hippocampus [22–24]. Regarding the role of the beta-band, these studies have shown that decreases of beta-band power are associated with movement execution in other brain areas such as the sensorimotor cortex and STN [25, 26]. Zhang *et al* also observed increases of beta-band power in primate somatosensory and motor cortices during movement inhibition [25]. The beta-band has also been linked to motor system inhibition in pathologic conditions such as Parkinson’s disease [27, 28]. For example, Kühn *et al* observed increases of beta-band power associated with motor inhibition in the STN in Parkinson’s disease patients performing Go/No-go tasks [26]. These findings have positioned the beta-band as a target in the development of aDBS therapies for Parkinson’s disease [29, 30].

In our previous study, we examined neural activity during direct arm-reaching movements (ARMs) and showed that hippocampal beta-band power decreases from baseline levels during the execution of movement [22]. In another previous study, we examined differences between motor execution and inhibition in a Go/No-go task, in which a green visual cue instructed the participant to initiate movement during ‘Go’ trials and a red visual cue to withhold movement during ‘No-go’ trials [23]. We observed that hippocampal beta-band power decreased below baseline during movement execution in ‘Go’ trials and increased above baseline during motor inhibition in ‘No-go’ trials, indicating that beta-band power is highly modulated during motor control [23].

The importance of these biological markers extends beyond clinical applications to include their utility as a source signal for brain-computer interfaces (BCIs), consisting of devices that use neuronal action potentials recorded by implanted electrodes to provide volitional control over external devices such as a robotic limb, motorized wheelchair, or cursor on a screen [31, 32]. Since recording high quality and consistent single-unit individual neuron activity is functionally challenging [33–35], several studies have explored local field potentials (LFPs) and found that they correlate with single-unit activity and can be used to extract and decode information [31, 36, 37]. Applying various signal processing modalities to LFPs can further facilitate the extraction and decoding of features by simplifying unstructured information within LFPs. For instance, principal component analysis (PCA) computes a linear transformation of data into a new orthogonal coordinate system, in which the coordinate dimensions represent decreasing amounts of variance. Thus, the first *N* principal components may capture the majority of the data’s variance while also reducing dimensionality and multi-collinearity [38]. Such decoding technique using principal component-based features has shown high accuracy [39–41].

Discriminant analysis (DA) is another popular technique that allows for dimension-reduction by capturing optimal combinations of data features [42]. DA may also be used as a classifier algorithm to maximize separability among identified classes in data [43], serving as a supervised form of learning compared to PCA [44]. Linear discriminant analysis (LDA) and quadratic discriminant analysis (QDA) are two particular types of DA examined in this study. LDA is typically used when datasets have close to equal covariance [45] and classifies based on distance from a linear line [46]. On the other hand, QDA allows for different covariance matrices [45] and identifies a quadratic fit to the data itself [47]. Both LDA and QDA have been previously used to decode neural signals during motor control in BCI applications such as in the P300 speller [48], to interpret EEG data [49] and motor imagery data [50], and in fMRI studies [51]. Recently, PCA has been used in conjunction with DA to reduce computational complexity and minimize the generalization error, a combination that is more effective than either method alone [44, 52]. For example, Onishi and Natsume successfully used PCA and DA for a P300-based BCI and concluded that this model showed better classification performance for BCI systems [53]. Both techniques have been utilized in studies specifically to decode neural signaling as well as in a wide range of other disciplines [54, 55].

The primary aim of this study is to assess the accuracy of five distinct discriminant models, integrated with a pre-processing stage using PCA, to discern between ‘Go’ and ‘No-go’ trials in a Go/No-go ARM task. Notably, while PCA and DA techniques have demonstrated their effectiveness in reducing data dimensionality and constructing robust models, the novelty of our approach lies in their application within the domain of a Go/No-go task utilizing beta-band (13–30 Hz) power samples of LFPs obtained from SEEG electrodes implanted in the hippocampus. Our study presents a focused approach that harnesses these established techniques within a distinct neurophysiological context, potentially bearing implications for advancements in BCI systems.

## 2. Methods

### 2.1. Participants

The data analyzed in this study were obtained from our previous experiment reported in Martin del Campo Vera *et al* [23]. The study participants included 10 participants (5 female and 5 male, ages between 21 and 46 years with a mean of 33.7 years) who were diagnosed with drug-resistant epilepsy. These participants were implanted with intracranial electrodes as part of their medical treatment for seizure localization. The number and placement of electrodes were customized to each participant based on MRI, positron emission tomography (PET) scans, video monitoring, and seizure semiology. Nine participants were implanted bilaterally in the hippocampus, whereas one participant (ID 8) was implanted only in the right hemisphere. Each case was discussed with neuroradiologists, epileptologists, and neurosurgeons at the University of Southern California (USC) Comprehensive Epilepsy Center Multi-disciplinary Conference. Table 1 provides a summary of participant profile information, including participant ID, age, gender, handedness, pathology, and seizure onset zone. Table 2 contains a detailed description of the electrodes implanted in the participants. All participants provided informed consent to participate in this study (Study ID: HS-17-00554), approved by the Institutional Review Board (IRB) of USC, Health Sciences Campus.

**Table 1.** Participant profiles: Profiles of the ten participants with drug-resistant epilepsy enrolled in the study who underwent stereotactic depth (intracranial) electrode implantation as part of their evaluation for seizure localization. The table provides an overview of their clinical characteristics relevant to the study, including information on age, gender, handedness, anatomic findings, and seizure-onset zones for each participant.

ID	Gender	Age	Handedness	Anatomic findings	Seizure-onset zone
1	F	45	Right	N/A	Right insula and frontal operculum
2	F	40	Right	N/A	Not localized
3	M	37	Left	Bilateral medial temporal sclerosis	Right and left temporal lobes
4	F	46	Right	N/A	Right orbitofrontal
5	F	21	Right	N/A	N/A
6	F	21	Right	Right parahippocampal gyrus cavernous malformation	Right anterior hippocampus
7	M	22	Left	Right hippocampal mesial sclerosis	Right amygdala
8	M	35	Right	N/A	Anterior Cingulate, Superior Temporal, Premotor, Posterior Central, Anterior & Posterior Resection (all in right hemisphere)
9	M	31	Right	N/A	Hippocampus Tail & Anterior Insula (all in right hemisphere)
10	M	39	Right	N/A	Mesial Temporal, Hippocampus & Amygdala (all in left hemisphere)

**Table 2.** Implanted Electrodes: Total number of electrodes implanted per patient, organized by cerebral hemisphere (left/right) and within specific anatomical regions of the hippocampus (anterior/posterior). The last column specifies the number of contacts located in the hippocampus, verified by pre-operative MRI and post-operative computed tomography (CT) imaging.

Participant ID	Cerebral hemisphere	Number of electrode leads implanted ^*^	Number of hippocampal contacts
1	Left	2 (1 AH, 1 PH)	7 (3 AH, 4 PH)
	Right	2 (1 AH, 1 PH)	7 (3 AH, 4 PH)
2	Left	2 (1 AH, 1 PH)	6 (3 AH, 3 PH)
	Right	2 (1 AH, 1 PH)	6 (3 AH, 3 PH)
3	Left	1 (AH)	3
	Right	1 (AH)	4
4	Left	2 (1 AH, 1 PH)	7 (4 AH, 3 PH)
	Right	2 (1 AH, 1 PH)	8 (4 AH, 4 PH)
5	Left	2 (1 AH, 1 PH)	10 (5 AH, 5 PH)
	Right	2 (1 AH, 1 PH)	9 (4 AH, 5 PH)
6	Left	1 (AH)	3
	Right	2 (1 AH, 1 PH)	9 (5 AH, 4 PH)
7	Left	1 (AH)	4
	Right	2 (1 AH, 1 PH)	3 (PH)
8	Right	1 (AH)	3
9	Left	1 (AH)	2
	Right	2 (1 AH, 1 PH)	2 (AH)
10	Left	2 (1 AH, 1 PH)	6 (3 AH, 3 PH)
	Right	1 (AH)	3
**AVG**	**Left (9 participants)**	**1.6**	**5.3 (3.3 AH, 3.6)**
	**Right (10 participants)**	**1.7**	**5.4 (3.4 AH, 3.8 PH)**

^*^ AH: Anterior Hippocampus, PH: Posterior Hippocampus.

### 2.2. Signal acquisition and pre-processing

Depth electrodes with 10 macro and 6 micro platinum contacts (MM16A-SP05X-000, Ad-Tech Medical Instrumentation Corporation, Oak Creek, WI, USA) were implanted for clinical treatment of epilepsy, with number and location of implanted electrodes determined solely by clinical need. No electrodes were implanted for only research purposes. The signals used in the present study are from the macro-type electrode contacts. Neural signals were amplified with unity gain, filtered with Butterworth 1st order analog high-pass (0.3 Hz) and 3rd order analog low-pass (7500 Hz) filters, and digitized with 16-bit, 250-nV resolution at 30 000 samples/sec using a Neural Signal Processor (NeuroPort System, Blackrock Microsystems, Salt Lake City, UT, USA) with built-in adaptive line (60 Hz) noise cancellation filter and 4th order hi/lo pass digital filtering for all channels, with the online reference contact located in white matter. Downsampling was then applied to the raw data sampled at 30 000 samples per second. First, a 4th order Butterworth low-pass filter with a cutoff frequency of 500 Hz was applied to the raw data to serve as an anti-aliasing filter. Subsequently, the data was subsampled (decimated) from 30 000 samples per second to 2000 samples per second. This down-sampled version was utilized in this analysis.

### 2.3. Experimental design: direct reach ‘center-out’ Go/No-go task

**Figure 1.**
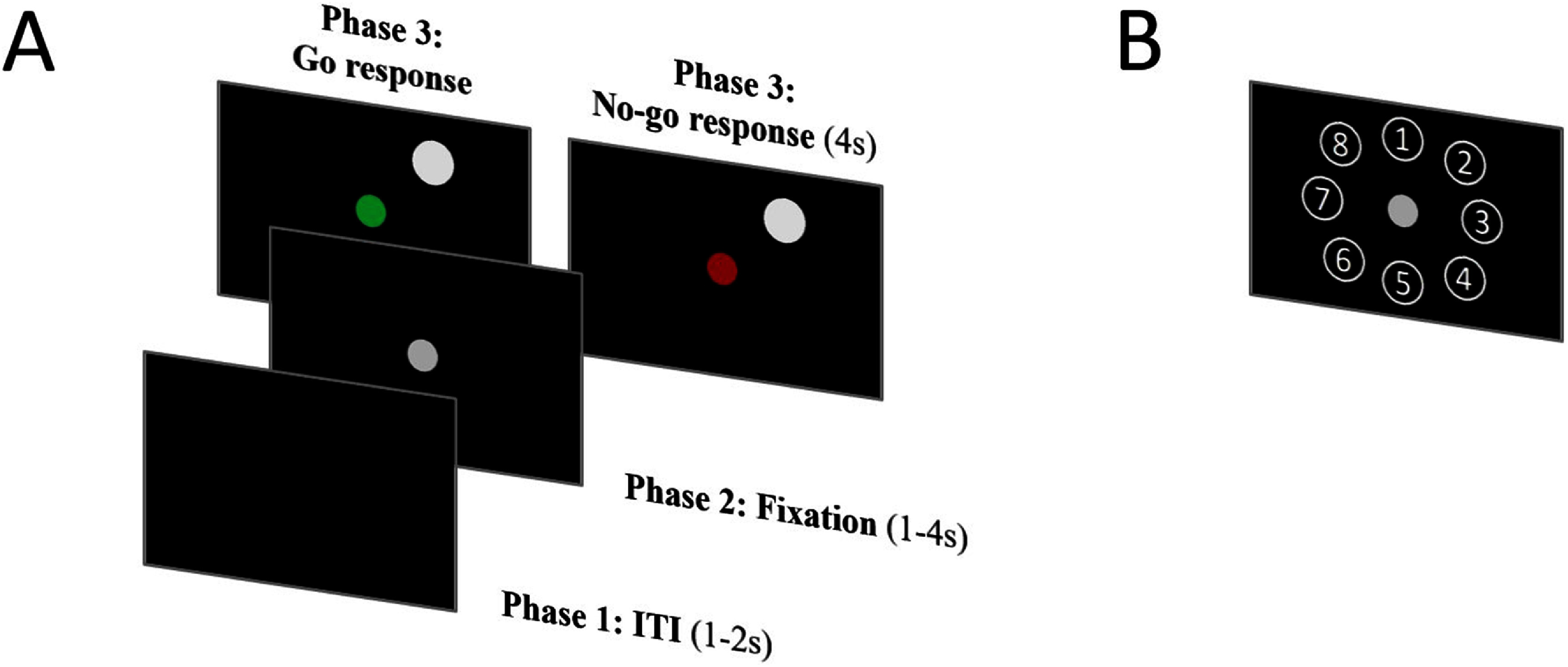
(A) Phase design of the experimental ‘Center-out’ direct-reach Go/No-go task. During the inter-trial interval phase (ITI), participants wait and prepare their hands at the center of the screen (1-2 s). During the fixation phase, a fixation dot appears in the center of the screen, participants are instructed to fixate their gaze and finger half an inch away from the fixation dot (1–4 s). During the response phase, participants are cued to either reach a target or withhold their movement (4 s) by turning the fixation dot green or red respectively. (B) Target locations for the experimental Go/No-go ‘Center-out’ direct reach task.

In this study, we used our previous variant of the classic ‘center-out’ direct-reach task that included two response conditions: ‘Go’ and ‘No-go’ [23]. The task was programmed in MATLAB© (2018b, The MathWorks, Inc., Natick, Massachusetts, USA) with the Psychophysics Toolbox Version 3 (PTB-3) and was presented on a 21.5 inch LED-backlit touchscreen monitor with 1920 × 1080 pixels and 250 cd/m2 luminance (S2240Tb, Dell Inc., Round Rock, TX, USA). The Go/No-go task consisted of 3 phases: ITI, Fixation, and Response (figure 1(A)). During the ITI phase that lasted 1–2 s, there was no visual cue on the screen, and the participants were tasked with holding their right hand approximately 2 inches away from the center of the screen and waited for the next phase to be displayed. Participants were then instructed to point to a gray fixation dot (9.53 mm radius) that displayed at the center of the touchscreen and to fix their gaze on the dot during the fixation phase (1–4 s). Phase lengths were varied to prevent participants from becoming accustomed to the movement task and to avoid timed responses. Participants were not moving their arms during this phase, which serves as a baseline period to measure modulations of the beta-band power spectrum against the subsequent response phase. Participants were monitored and recorded with a video camera to ensure that there was no movement during the Fixation and ‘No-go’ response phases.

The response phase was split into two different conditions. During the ‘Go’ condition, a white circle (15.88 mm radius) appeared in pseudo-random order at one of the eight target locations equidistant around the center fixation dot, 114.3 mm center to center (figure 1(B)). Simultaneously with the display of the target, the fixation dot turned green to cue the participants to move toward the target. The participants were instructed to move their right arm toward the target to double-tap the screen at the location of the target as soon as the target and the green cue were displayed. The response time was defined as the time difference between the target appearance and the participant’s double-tap. During the ‘No-go’ condition, the same white circle appeared pseudo-randomly at one of the same eight target locations as in the ‘Go’ condition. However, the fixation dot turned red instead of green to cue the participants to not move toward the target and remain in the holding position on the fixation dot. The time duration of the ‘No-go’ condition was 4 s. For the entire response phase, a total of 64 trials (8 trials per target location) were conducted for each participant, with 32 trials having the ‘Go’ condition and the other 32 having the ‘No-go’ condition.

### 2.4. Criteria and considerations for data analysis

Data was recorded from electrode contacts located in the anterior and posterior hippocampal gray matter, identified from merged preoperative MRI and postoperative CT scans, were included for analysis in this study. Information about the number of leads implanted, hippocampal gray matter contacts, and targeted brain regions for each participant is provided in table 2.

Trials were manually inspected for IISs. Participants who presented IISs in 50% of the trials or more were removed from the analysis (only one participant was excluded for this reason). In addition, the following trials were removed from the analyses: (1) trials in which participants moved during the Fixation and ‘No-Go’ response phases, (2) instances where the participant did not double tap successfully on the target, (3) responses with a duration of less than 200 ms, which were considered accidental (none of the trials in this study had response times less than 200 ms, with the shortest minimum response time being 0.83 s from participant 1), and (4) instances where power values were identified as outliers according to the IQR rule, that is, measures with power values more than 1.5 times the IQR above the 3rd quartile or below the 1st quartile were excluded from the analyses. No responses with movement disorder aspects or abnormal behaviors outside the scope of the ARMs were observed. Table 5 presents the patient performance with the proportion of successful trials after removing failed trials.

### 2.5. Spectral power analysis

Power spectral densities were computed using the multitaper method due to its anti-leakage properties that provide high frequency resolution [56] with Chronux (Woods Hole, MA) [57] in MATLAB© 2018b. Given a set of *N* orthogonal tapers, the multitaper method computes the Fourier transform of *N* copies of the same data segment, each having one of the tapers applied. By averaging these results to produce a single estimate of the power spectral density, the multitaper method produces a power spectrum with reduced variance and spectral leakage [58]. This method was particularly advantageous for our analysis because the use of multiple tapers facilitates the effective isolation of the beta (13–30 Hz) band power from adjacent frequencies. We computed the multitaper power spectral with 9 leading tapers and a time-bandwidth product of 5.

LFP data from each phase of the experimental task were trimmed by a variable amount between 0.1 and 1.75 s at the beginning and end to exclude the transition between phases and to retain 0.5 s of data for analysis. In the case of the ITI, Fixation, and ‘No-go’ Response phases, the window used was centered in the middle of these phases; whereas, for the ‘Go’ Response phase, the window was aligned to the end of the phase to capture a more stereotyped part of the reach (the time just before tapping on the screen).

### 2.6. Principal component and cluster analysis

PCA was performed to reduce the complexity and dimensionality of beta-band power modulation from multiple channels and multiple frequency bins within the beta-band into two multiple components [38]. By identifying high variance dimensions within the neural data, PCA allowed us to extract the contribution from channels to the modulation that best distinguishes the conditions of our task. Data from the left and right hippocampi were appended into a single two-dimensional matrix used as input to the PCA and projected onto the first two principal component coordinates in preparation for cross-validated classification accuracy testing (see section 2.7). Additionally, the data points transformed into the new coordinate system defined by the first two principal components were utilized for cluster analysis.

PCA was specifically performed on data obtained exclusively during the response phase of the experimental task, which is central to our study’s goal of classifying ‘Go’ and ‘No-go’ responses. This phase was selected due to its direct relevance to the execution and inhibition of arm movements, in line with our objective to investigate movement-related beta-band power in hippocampal electrophysiological oscillations. Therefore, our emphasis on the response phase ensures that our classification analysis is precisely focused solely on stereotyped responses associated with the ‘Go’ and ‘No-go’ conditions.

The Silhouette score, introduced by Rousseeuw in [59], was used for cluster analysis to assess the quality of clustering of data points between the ‘Go’ and ‘No-go’ conditions. The Silhouette score (calculated using the Matlab function ‘silhouette’) measures how closely a data point resides within its own cluster (cohesion or tightness), as well as how distinct the same data point is from other clusters (separation). The Silhouette score ranges from −1 to +1, with values closer to +1 indicating data points that are more similar to its own cluster than to others, values closer to −1 indicating data points that are more similar to other clusters than to their own cluster, and values closer to 0 indicating data points that are close to the decision boundary between clusters.

### 2.7. Cross-validated DA classification

The accuracy of several classification models was tested using 10-fold cross-validation (CV) [60]. This statistical method involves the random partitioning of data into *k* subsets, where *k* is set to 10 in this study. One of these subsets is used to validate the model, and the remaining subsets are used to train the model. This process repeats once for each partition of the data. Five different models were considered for the DA classifiers: Linear, Diagonal-Linear, Pseudo-Linear, Diagonal-Quadratic, Pseudo-Quadratic.

The cross-validated error rate, which is the ratio of wrong decisions to the total number of cases, was calculated using the Matlab function ‘kfoldloss.’ Since the function randomly shuffles and samples the dataset, each run computes different error rates for each classification model. To account for the variability in these error rates, the 10-fold CV was performed 10 times, with each repetition involving a different random partitioning of the data. This choice of conducting 10 repetitions aims to account for the potential variations in model performance. This approach is typically applied in statistical learning [61–64]. Across all participants, the error rates obtained from these 10 repetitions were averaged to provide a more robust assessment of model performance. A similar computational method was conducted for standard errors, and all participant estimates are listed in table 4.

## 3. Results

### 3.1. Principal component and cluster analysis

**Figure 2.**
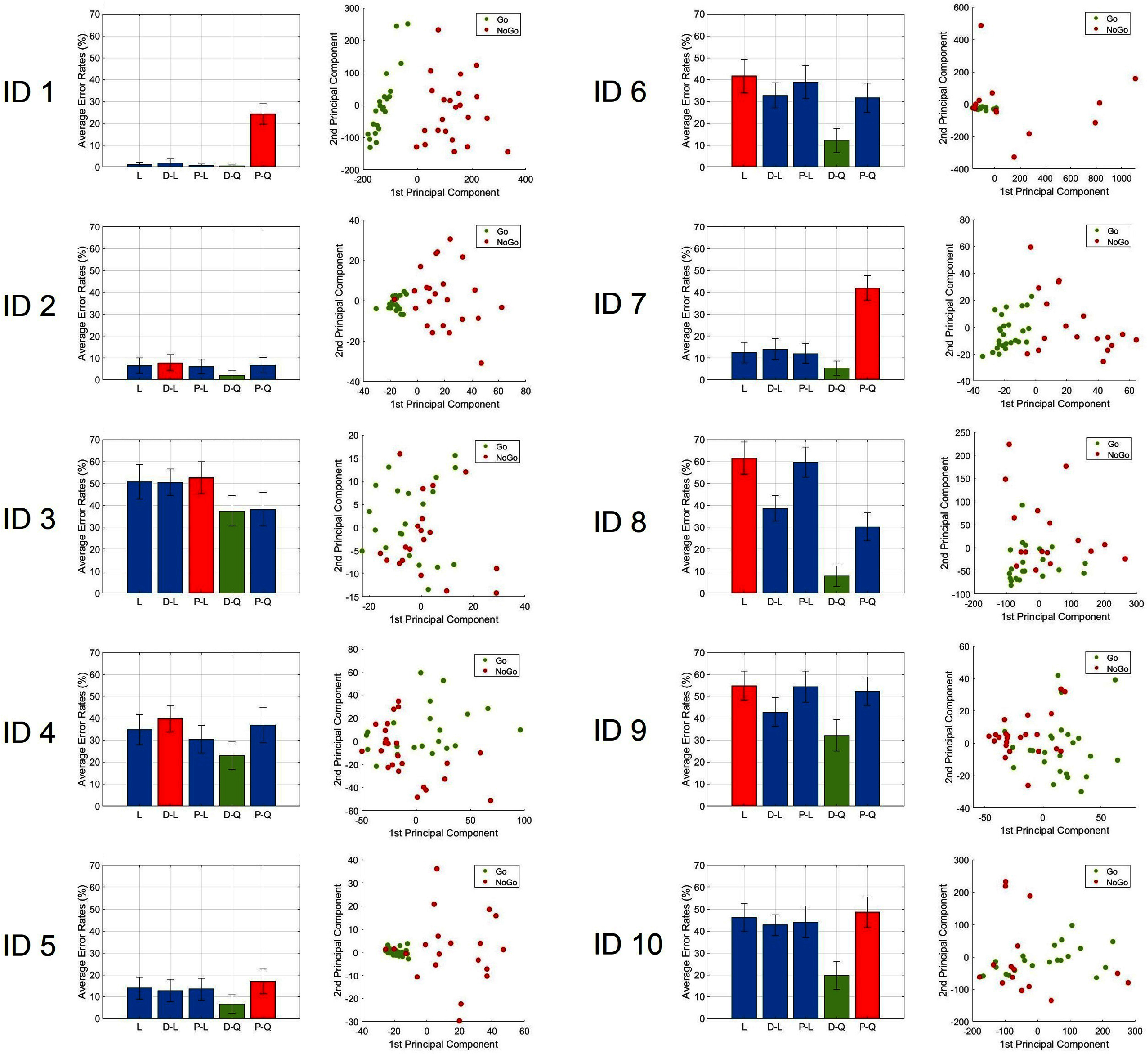
Left panel: Average error rates from the five tested models in all participants, the best performing model with the lowest error rate is highlighted in green; the worst performing model with the highest averaged error rate is highlighted in red. Error bars represent standard deviations. (L: Linear model, D-L: Diagonal-Linear model, P-L: Pseudo-Linear model, D-Q: Diagonal-Quadratic model, P-Q: Pseudo-Quadratic model). Right panel: Clusters extracted by principal component analysis from all participants.

The first two principal components (second column of figure 2) accounted for 54.8% (range: 36.9% in participant 4%–81.3% in participant 6) of the total variance explained, averaged across all participants. The PC1 accounted for 40.2% (range: 24.3% in participant 4%–71.1% in participant 6) of the total variance explained, averaged across all participants. The PC2 accounted for 14.6% (range: 7.9% in participant 5%–29.8% in participant 8) of the total variance explained, averaged across all participants.

**Figure 3.**
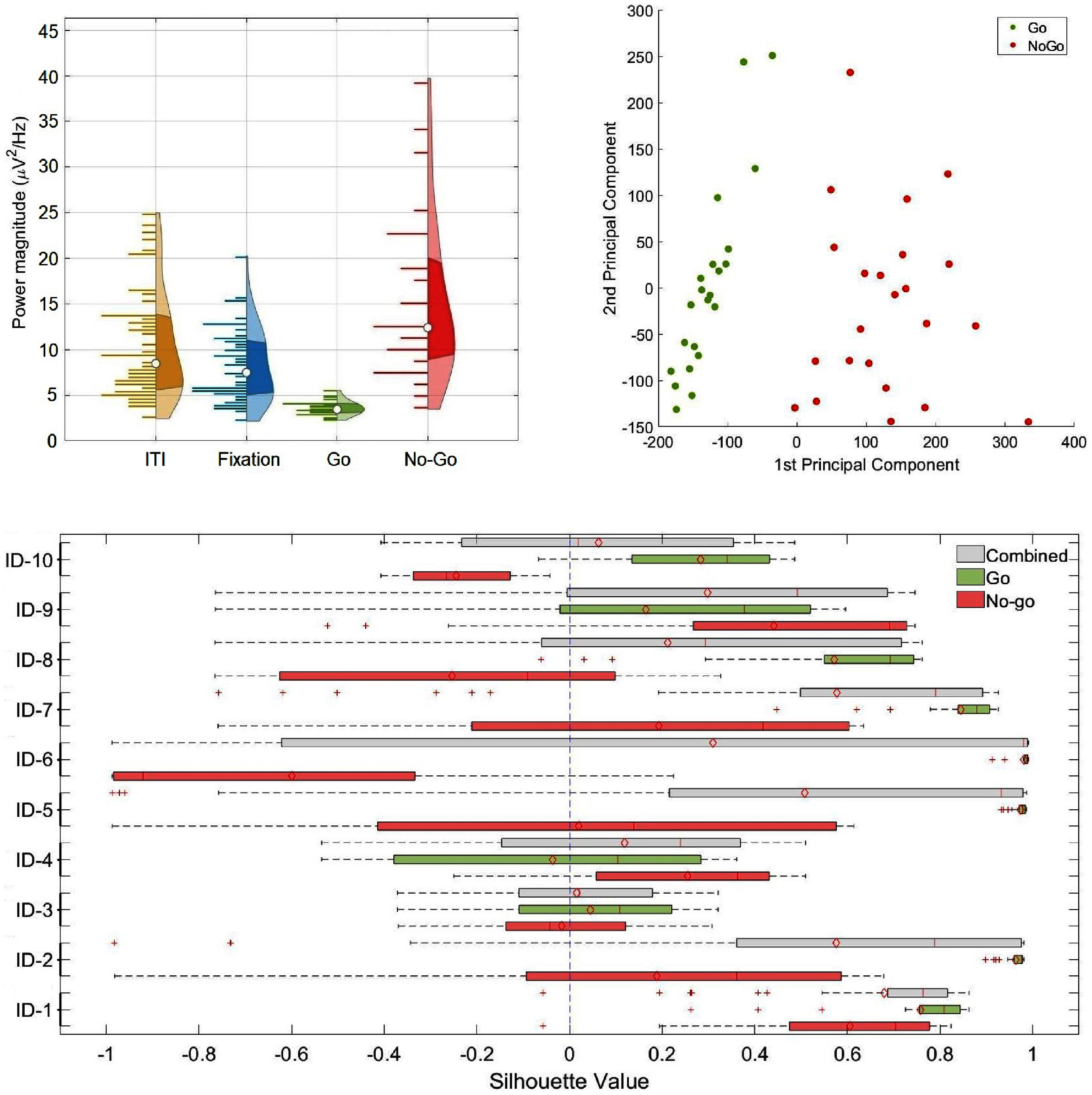
Top-left panel: Averaged beta-band (13–30 Hz) power spectral density half-violin plots. During the pre-processing stage, beta-band power magnitude measures from hippocampal contacts were used for both the ‘Go’ and ‘No-go’ conditions as input features. Top-right panel: Clusters were extracted by principal component analysis from all frequency bins within the beta-band power (13–30 Hz) and gray-matter contacts in the hippocampus, the first two principal components were used for cross-validated DA. Bottom panel: Silhouette score box plots from all participants grouped by ‘Combined’ (gray), ‘Go’ (green), and ‘No-go’ (red) trials.

Graphs from each of the participants exhibited a degree of clustering of data points by experimental condition (‘Go’ versus ‘No-go’), with the graph for participant ID 1 showing no visual overlap between the clusters of both conditions (top-right panel of figure 3). Furthermore, five additional participants (IDs 2, 4, 5, 7, and 8) exhibited visually distinct clusters between ‘Go’ and ‘No-go’ trials with some overlap, while the remaining participants (IDs 3, 6, 9, and 10) displayed a greater overlap between the clusters, making them less visually distinguishable (second column of figure 2).

Mean and median Silhouette scores were computed for each participant, both when considering all trials together (to evaluate individual responses without regard of response condition), and when considering ‘Go’ and ‘No-go’ trials separately (to assess individual responses in relation to response condition).

When evaluating all trials combined, the mean and median Silhouette scores for all participants were greater than zero. Box plots, showing the distribution of Silhouette scores for individual participants, can be found in the bottom panel of figure 3 (gray box plots).

When evaluating the ‘Go’ and ‘No-go’ trials separately, for ‘Go’ trials, 9 out of 10 participants had mean Silhouette scores above 0, and all participants had median Silhouette scores greater than 0. In the case of ‘No-go’ trials, 6 out of 10 patients achieved mean and median Silhouette scores above 0. The distribution of Silhouette scores for each participant under the Go/No-go conditions is depicted in the bottom panel of figure 3 (green and red box plots). The specific values of mean and median Silhouette scores for each participant are provided in table 3.

**Table 3.** Evaluation of Go and No-Go clusters: Silhouette scores for ‘Go’ and ‘No-go’ task conditions for each participant, detailing mean and median values for each task condition and for both conditions combined. The final two rows display the average and median silhouette scores across all participants for each task condition and for both conditions combined.

Participant ID	Silhouette scores					
	Go trials		No-Go trials		Go and No-Go trials combined	
	Mean	Median	Mean	Median	Mean	Median
1	0.76	0.81	0.60	0.70	0.68	0.76
2	0.96	0.98	0.19	0.36	0.58	0.79
3	0.05	0.11	−0.02	−0.04	0.02	0.02
4	−0.04	0.10	0.26	0.36	0.12	0.24
5	0.97	0.98	0.02	0.14	0.51	0.93
6	0.98	0.99	−0.60	−0.92	0.31	0.98
7	0.84	0.88	0.19	0.42	0.58	0.79
8	0.57	0.69	−0.25	−0.09	0.21	0.29
9	0.17	0.38	0.44	0.69	0.30	0.49
10	0.28	0.34	−0.24	−0.27	0.06	0.02
**AVG**	**0.55**	**0.63**	**0.06**	**0.14**	**0.34**	**0.53**
**MEDIAN**	**0.67**	**0.75**	**0.11**	**0.25**	**0.31**	**0.63**

### 3.2. Cross-validated DA classification

Principal components computed above were used as features to classify the beta-band power between the ‘Go’ and ‘No-go’ conditions. The performance of the five different models examined in this study was compared using 10-fold cross-validated error rates (equivalent to 1—the classification accuracy rate). Table 4 reports the average 10-fold cross-validated error rates across 10 runs, along with their corresponding standard error, for the five different types of DA classifiers tested in each participant.

Among all classification models, the Diagonal-Quadratic model provided the lowest cross-validated classification error rate across all participants, averaging 14.7% and ranging from 0.4% (good) in participant 1%–37.6% (poor) in participant 3. The Diagonal-Quadratic model also provided the lowest standard error in the majority of participants (6 out of 10), and the lowest averaged standard error across all participants (average 4.7, range 0.4-7.1) compared with the other models (second-to-last row of table 4). Thus, this model was found to be the optimal model for this cross-validated DA among the cohort of participants in the study (table 4).

**Table 4.** Performance of classification models: Performance metrics of classification models across participants, detailing the proportion of variance explained by the first two principal components for each participant and the classification error rates, along with their standard errors, for each tested classification model. The models include Linear, Diagonal-Linear, Pseudo-Linear, Diagonal-Quadratic, and Pseudo-Quadratic. The final two rows present the average and median values of these metrics across all participants. Notably, the Diagonal-Quadratic model yielded the lowest classification error rates across all models tested, both at the individual and group levels (highlighted in gray).

Participant ID	Proportion of variance explained		Classifier method	Classification error (%)	Standard errors for K-fold cross validation (%)
	PC1 (%)	PC2 (%)			
1	35.25	17.00	Linear	1.11	1.05
			Diag-Linear	1.78	1.95
			Pseudo-Linear	0.67	0.60
			Diag-Quadratic	0.44	0.40
			Pseudo-Quadratic	24.22	4.65
2	49.43	11.40	Linear	6.52	3.56
			Diag-Linear	7.83	3.67
			Pseudo-Linear	6.09	3.29
			Diag-Quadratic	2.17	2.20
			Pseudo-Quadratic	6.74	3.60
3	25.76	13.18	Linear	50.73	7.87
			Diag-Linear	50.49	6.04
			Pseudo-Linear	52.68	7.32
			Diag-Quadratic	37.56	7.03
			Pseudo-Quadratic	38.29	7.66
4	24.29	12.63	Linear	34.67	6.89
			Diag-Linear	39.78	6.00
			Pseudo-Linear	30.44	6.26
			Diag-Quadratic	22.89	6.20
			Pseudo-Quadratic	36.89	8.14
5	38.91	7.89	Linear	13.90	5.09
			Diag-Linear	12.69	5.11
			Pseudo-Linear	13.42	5.10
			Diag-Quadratic	6.59	4.13
			Pseudo-Quadratic	17.07	5.57
6	71.05	10.20	Linear	41.51	7.68
			Diag-Linear	32.72	5.66
			Pseudo-Linear	38.78	7.65
			Diag-Quadratic	12.12	5.49
			Pseudo-Quadratic	31.51	6.69
7	38.30	17.31	Linear	12.50	4.78
			Diag-Linear	14.09	4.85
			Pseudo-Linear	12.05	4.57
			Diag-Quadratic	5.46	3.26
			Pseudo-Quadratic	42.04	5.60
8	50.94	29.83	Linear	61.54	7.35
			Diag-Linear	38.72	5.88
			Pseudo-Linear	59.74	6.89
			Diag-Quadratic	7.69	4.66
			Pseudo-Quadratic	30.26	6.41
9	36.75	12.49	Linear	54.79	6.81
			Diag-Linear	42.71	6.53
			Pseudo-Linear	54.38	7.09
			Diag-Quadratic	32.08	7.14
			Pseudo-Quadratic	52.29	6.56
10	31.76	13.94	Linear	46.11	6.48
			Diag-Linear	42.78	4.69
			Pseudo-Linear	44.17	7.22
			Diag-Quadratic	19.72	6.40
			Pseudo-Quadratic	48.61	6.94
**AVG**	**40.24**	**14.59**	**Linear**	**32.34**	**5.76**
			**Diag-Linear**	**28.36**	**5.04**
			**Pseudo-Linear**	**31.24**	**5.60**
			**Diag-Quadratic**	**14.67**	**4.69**
			**Pseudo-Quadratic**	**38.23**	**6.18**
**MEDIAN**	**37.53**	**12.91**	**Linear**	**38.09**	**6.645**
			**Diag-Linear**	**35.72**	**5.385**
			**Pseudo-Linear**	**34.61**	**6.575**
			**Diag-Quadratic**	**9.905**	**5.075**
			**Pseudo-Quadratic**	**34.2**	**6.485**

The averaged 10-fold CV results for all models in all participants are shown in table 4. For each participant, the best performing model with the lowest average cross-validated error rate has been highlighted in green (Diagonal-Quadratic model in 10 participants). The worst performing model with the highest average cross-validated error rate has been highlighted in red (Pseudo-Quadratic model in 4 participants, Linear model in 3 participants, Diagonal-Linear in 2 participants, and Pseudo-Linear in 1 participant).

Finally, mean reaction times for each participant during the experimental paradigm are detailed in table 5, including the proportion of successful trials for both the ‘Go’ and ‘No-go’ tasks. At the group level, the mean reaction time averaged across all participants was 1.59 s, with a standard deviation of 0.31 s (table 5). The success rate averaged across all participants was 97.2% for the ‘Go’ condition and 90.9% for the ‘No-go’ condition, respectively.

**Table 5.** Participant performance metrics: Summary of performance metrics for each participant, including mean reaction times for ‘Go’ trials (from cue presentation to motor response), standard deviation of reaction times, and the proportion of successful trials for both ‘Go’ and ‘No-go’ conditions. Trial success was determined based on the criteria outlined in section 2.4. The last row presents the average performance metrics across all the participants.

Participant ID	Mean reaction time (seconds)	Standard deviation of reaction times (seconds)	Proportion of successful trials	
			Go	No-go
1	1.24	0.44	31/32	32/32
			(96.9%)	(100.0%)
2	2.22	0.49	32/32	32/32
			(100.0%)	(100.0%)
3	1.54	0.29	31/32	32/32
			(96.9%)	(100.0%)
4	1.79	0.25	31/32	32/32
			(96.9%)	(100.0%)
5	1.62	0.42	29/32	32/32
			(90.6%)	(100.0%)
6	1.17	0.14	31/32	27/32
			(96.9%)	(84.4%)
7	1.42	0.19	32/32	25/32
			(100.0%)	(78.1%)
8	2.01	0.29	31/32	23/32
			(96.9%)	(71.9%)
9	1.53	0.25	32/32	31/32
			(100.0%)	(96.9%)
10	1.34	0.30	31/32	25/32
			(96.9%)	(78.1%)
**AVG**	**1.59**	**0.31**	**31.1/32**	**29.1/32**
			**(97.2%)**	**(90.9%)**

## 4. Discussion

In this study, we implemented an offline approach that utilized PCA from hippocampal beta-band power as the source feature for DA between ‘Go’ and ‘No-go’ trials in a Go/No-go task. This approach reduced data dimensionality from multiple frequency bins within the beta-band and multiple hippocampal contacts into two principal components. Subsequently, we assessed the error rates of five cross-validated DA classification models based on these components. The objective of our study was to design an approach using PCA to streamline the complexity of beta-power modulation within the hippocampus coupled with DA to classify Go/No-go arm-reaching responses, potentially for use in BCI applications. While PCA and DA are established methods, the novelty of our approach lies in its application to classify motor execution and inhibition in Go/No-go arm-reaching tasks from the human hippocampus. PCA is known for reducing multi-collinearity and simplifying data, making it suitable for BCI applications, for example, it has been used to avoid redundancy of features in an EEG-based BCI system for classification of self-induced emotions [65]. In addition, PCA and DA have also been successfully applied in several cases to simplify data input for BCI applications, a finding that motivated our current study [53].

All participants exhibited a relative increase of median beta-band power associated with movement inhibition during ‘No-go’ trials compared with the Fixation (baseline), and a relative decrease in median beta-band power associated with movement response during ‘Go’ trials (top-left panel of figure 3), supporting the involvement of hippocampal beta-band power in the execution or inhibition of voluntary movements [22, 23]. Interestingly, in a related study by Gilbert *et al*, the reaction times of ‘Go’ trials were found to be significantly higher when compared to an ‘Always-Go’ task, highlighting the impact of the ‘Go/No-go’ uncertainty conditions on participant response times [24]. In addition, this observation aligns with other groups’ studies that have shown beta-band power as a reliable marker of movement onset and offset in the sensorimotor cortex [66–68].

While the majority of participants displayed Silhouette scores above zero during ‘Go’ trials (9 participants based on the mean Silhouette score and 10 based on the median), the distribution of Silhouette scores for the ‘No-go’ trials displayed fewer participants with mean and median values above zero, with 4 participants exhibiting negative mean and median Silhouette scores in the ‘No-go’ trials (IDs 3, 6, 8, and 10). Apart from a greater number of participants displaying negative or near-zero mean and median Silhouette scores in the ‘No-go’ condition, another difference between the ‘Go’ and ‘No-go’ trials is the higher variability observed from the box plots of the ‘No-go’ condition (bottom panel of figure 3, green and red box plots), leading to a more scattered arrangement of data points within the ‘No-go’ condition (e.g. IDs 2, 5, 6, 7, and 8 from figure 2).

Furthermore, the 4 participants (IDs 3, 4, 9 and 10) with the highest classification error rate for the Diag-Quadratic model (37.6%, 22.9%, 32.1%, and 19.7%, respectively) also presented negative or near zero mean and median Silhouette scores. This finding may be related to the location and number of electrodes placed in the hippocampus. 3 out of 4 of the above participants had fewer implanted contacts than the others (7 for ID 3, 4 for ID 9, and 9 for ID 10). By contrast, participant 1, who showed relatively high mean and median Silhouette scores in comparison to overall group-level performance and exhibited the lowest classification error rate of 0.4%, had relatively higher number of implanted electrodes (14). One possible explanation for this finding may be related to inclusion of electrode contacts that do not display beta-band modulation. In our previous study, we observed that in the same participants, several electrode contacts in the hippocampus did not display significant beta-band power modulation while others did [23]. This variability in the modulation of different channels within the hippocampus is likely due to anatomical variations or noise within individual channels. In this way, including non-significantly modulating channels in the analysis will likely decrease the accuracy of the classification model.

In assessing effectiveness of the five classification methods, we found that the Diagonal-Quadratic model was optimal given the lowest average error rates across all participants, especially in participants 1, 2, 5, 7, and 8, who had error rates below the median value of 9.9% (0.4%, 2.2%, 6.6%, 5.5%, and 7.7% respectively). This observation suggests that PCA paired with a Diagonal-Quadratic discriminant function can be an effective approach to discriminate between Go/No-go responses.

The higher accuracy of a Diag-Quadratic classification model over linear models suggests that a more complex relationships (e.g. nonlinear decision boundaries) in beta-band power modulation of motor control are likely to be present [69]. One possible explanation for this can be related to the 1/f (pink noise) nature of brain oscillations. In addition to oscillatory, or rhythmic activity, neural oscillations can be influenced by pink noise (1/f), in which the frequency spectrum is inversely proportional to the neural data. Because of this inverse relationship, neural oscillations at lower frequencies have a more pronounced decay than at higher frequencies, which may contribute to nonlinear attributes of neural data. In other words, lower-frequency oscillations may be more influenced by slow, nonlinear processes [70]. Arrhythmic pink noise specifically in the low-frequency ranges have been shown to modulate behavioral performance and decision-making [71, 72]. Aghababaiyan determined that while underlying pink noise can amplify the power spectral density measured from an neuron, the magnitude of this amplification was dependent on the frequency range [73]. In this way, they showed that pink noise affects neural signaling in a non-linear fashion [73]. Additionally, areas involved in motor control and decision making may exhibit complex, nonlinear dynamics, particularly in the field of epileptology, where EEG data is widely regarded as exhibiting non-linear dynamics. Andrzejak *et al* compared the accuracy of linear versus nonlinear time series analysis in locating hippocampal seizure onset zones in patients with epilepsy [74]. They determined that analytic techniques based on nonlinear dynamics were far superior in representing the spatiotemporal dynamics of epileptic activity [74]. Similarly, Guevara Erra *et al* measured MEG signals during a movement task and determined that a nonlinear model best represented the resulting electrophysiological signals, and posited that this was due to the compounded effects of decision making and movement processes [75]. In these ways, underlying neural circuitry, decision making, and motor action may reflect complex neurological processes that are better represented with non-linear metrics.

The higher accuracy achieved by the Diag-Quadratic model can also be an indicator that the assumptions of this particular model align more closely with the structure of our data. Specifically, the Diag-Quadratic model differentiates itself from other quadratic models due to its consideration of feature independence and underlying assumptions. This distinction is closely tied to the choice of the covariance matrix employed by the Diag-Quadratic model. The term ‘diag’ in Diag-Quadratic refers to diagonal covariance matrices. In practical terms, this means that the features within each class are assumed to be uncorrelated. In our case, the features correspond to the two principal components derived from beta-band power data after conducting PCA, whereas the classes represent the categories we aim to classify (Go/No-go). Consequently, if the data indeed exhibits a diagonal covariance structure, the Diag-Quadratic model is likely to outperform other models that have a different covariance structure. In contrast, linear models assume equal covariances across classes, which may not effectively capture the diagonal structures in the data.

Limitations of our study include a relatively small sample size of participants (*n* = 10), cohort heterogeneity, and the complexities associated with chronic epilepsy, which may limit the generalizability of these results. In addition, because the site of electrode placement was chosen based on clinical circumstances, electrode placement may not optimize for brain regions that would yield high classification accuracy. We also attempted to isolate the effects of the beta-band on movement and minimize confounds by using a center-out direct-reach Go/No-go experiment. Because we only observed an increase in beta-band power during physical movement and because participants were unaware of where the target would appear, the effects we observed are more likely associated with movement execution and less likely related to planning. However, we were unable to account for the effects of beta-band power on other confounds during response, such as eye movement.

We found previously that there was no significant difference in beta-band modulation when comparing right- and left-sided hippocampal contacts in the same participants in the same task [23]. Therefore, we did not include as separate analysis for right- vs. left-sided contacts in the current study. The pooling of contacts from both sides made for a more simple and robust analysis. It is possible that with a larger collection of participants, a significant difference in modulation between sides could be seen, so future studies with a larger number of participants could implement laterality into a classification model.

As discussed above, we believe that the relatively high error rates of the Diag-Quadratic model in some patients may reflect the presence of electrode contacts in the hippocampus that do not display significant beta-band modulation. Including non-significantly modulating channels may decrease the accuracy of the classification model, which could have implications in implementing this model in an on-line BCI system. It would, therefore, be necessary to develop a method to filter contacts that do not contribute to the accuracy of the model in real time. This could potentially be accomplished by performing a real-time analysis to assess the quality of the data from each contact and subsequently filter out those contacts with lower quality data. Future studies may involve implementing a task to identify higher quality contacts to be integrated in the BCI system. Other future research may include elucidating the function of the beta-band in motor intention in other brain areas that are part of the corticolimbic circuitry, such as the amygdala and orbitofrontal cortex. Analyzing the contributions of other brain areas could help to calibrate the classifier model and allow for a more accurate BCI.

Our study utilized data from the end of the response phase, when the reaching movements would be stereotyped across trials just before tapping, with the aim to classify between movement execution and inhibition. While this approach is valuable for decoding stereotyped responses, it may have a limitation in the form of lagged discrimination within real-time BCI systems. Future BCI research could consider early discrimination of intended actions, for instance, by employing delayed reach tasks. These tasks introduce a delay between cue presentation and response, offering an opportunity to dissect anticipatory movement planning from actual movement execution. Given that prior literature has shown beta de-synchronization during motor preparation [14, 25, 76], such experimental tasks may enhance BCI systems’ ability to decode ‘Go’ trials in a more anticipatory manner. This could potentially reduce any lag in signal extraction and improve the responsiveness of BCI systems.

In this study, our analysis was specifically focused on the beta frequency band (13–30 Hz), selected for its well-documented role in movement execution and inhibition [77–79]. This methodological decision aimed to assess whether the spectral power within the beta band alone could serve as a reliable source for classifiers to discriminate between ‘Go’ and ‘No-go’ trials. Notably, specific participants, such as IDs 1, 2, 5, 7, and 8, exhibited exceptionally low classification errors with the Diagonal-Quadratic classifier (0.44%–7.69% error rates), suggesting that the beta band alone can indeed provide substantial discriminative power. However, the variability and increased error rates observed among the rest of the participants, using the same classifier (12.12%–37.56% error rates), underline the potential benefits of incorporating a broader range of frequency bands. Such inclusion could potentially improve classification accuracy and reduce inter-participant variability by capturing a more comprehensive spectrum of movement-related information. In particular, expanding movement-related classification methods to encompass the gamma band (30–200 Hz) may present a compelling avenue for future investigations, as this band has also been implicated in various aspects of movement control, including the processing of motor commands and the integration of sensory feedback during movement, as well as in attention and working memory [80–83]. All of these roles further support its potential utility in enhancing the accuracy of movement-related classifications. Additionally, examining modulation in frequency bands could build upon the findings of the present study and deepen our understanding of the neural complexities of movement processing.

Overall, the findings of this study demonstrate that PCA paired with a Diagonal-Quadratic classification model can be an effective method for discriminating between Go/No-go trials from beta-band power in the hippocampus during ARMs, lending credence to the role of hippocampal beta-power modulation during motor control. Our findings not only contribute to our knowledge of neuromodulation mechanisms, but also have implications for the development of BCI applications and therapeutic interventions for patients with motor impairments.

## Data Availability

The data cannot be made publicly available upon publication because no suitable repository exists for hosting data in this field of study. The data that support the findings of this study are available upon reasonable request from the authors.
